# Do unfavourable working conditions explain mental health inequalities between ethnic groups?: cross-sectional data of the HELIUS study

**DOI:** 10.1186/s12889-015-2107-5

**Published:** 2015-08-20

**Authors:** Karen Nieuwenhuijsen, Aart H. Schene, Karien Stronks, Marieke B. Snijder, Monique H. W. Frings-Dresen, Judith K Sluiter

**Affiliations:** Coronel Institute of Occupational Health, Academic Medical Center (AMC), University of Amsterdam, P.O. Box 22700, Amsterdam, The Netherlands; Department of Psychiatry, Radboud University Medical Center, Reinier Postlaan 6, 6500 HB Nijmegen, The Netherlands; Donders Institute for Brain, Cognition and Behavior, Radboud University, Geert Grooteplein Noord 21, 6525 EZ Nijmegen, The Netherlands; Department of Public Health, Academic Medical Center (AMC), University of Amsterdam, P.O. Box 22700, Amsterdam, The Netherlands

## Abstract

**Background:**

Ethnic inequalities in mental health have been found in many high-income countries. The purpose of this study is to test whether mental health inequalities between ethnic groups are mediated by exposure to unfavourable working conditions.

**Methods:**

Workers (*n* = 6278) were selected from baseline data of the multi-ethnic HELIUS study. Measures included two indices of unfavourable working conditions (lack of recovery opportunities, and perceived work stress), and two mental health outcomes (generic mental health: MCS-12 and depressive symptoms: PHQ-9). Mediation of the relationships between ethnicity and mental health by unfavourable working conditions was tested using the bias-corrected bootstrap confidence intervals technique. Linear models with and without the mediators included, and adjusted for gender and age. Attenuation was calculated as the change in B between the models with and without mediators.

**Results:**

The sample comprised Dutch (1355), African Surinamese (1290), South-Asian Surinamese (1121), Turkish (1090), Ghanaian (729), and Moroccan (693) workers. After controlling for age and gender, all ethnic minorities had a higher risk of mental health problems as compared to the Dutch host population, with the exception of Ghanaians in the case of depressive symptoms, and African Surinamese workers with regard to both outcomes. The Turkish group stands out with the lowest mental health on both mental health indices, followed by Moroccan and South-Asian Surinamese workers. A lack of recovery opportunities mediated the relationship between ethnic group and a higher risk of mental health problems. Perceived work stress did not contribute to the explanation of ethnic inequalities.

**Conclusions:**

The higher risk of mental health problems in ethnic minority groups can be partly accounted for by a lack of recovery opportunities at work, but not by perceived work stress. This may imply that workplace prevention targeting recovery opportunities have the potential to reduce ethnic inequalities, but ethnic-specific experiences at the workplace need to be further explored.

## Introduction

Ethnic inequalities in mental health have been found in many high-income countries [1–5]. The Netherlands is no exception, with large mental health inequalities found between ethnic groups. A study of migrants in Amsterdam found the one-month prevalence of depressive and/or anxiety disorders to be higher in Turkish (18 %) and Moroccan (10 %) groups and lower in Surinam/Antillean (1 %) groups compared to Dutch (7 %) [6]. The higher levels of depressive symptoms in these groups are not likely to be explained by a higher tendency to emphasize symptoms given the findings of a study comparing Turkish and Moroccan ethnic groups with ethnic Dutch subjects. This study found that Turkish and Moroccan groups showed similar depressive symptom profiles despite their overall higher level depressive symptoms [7]. Moreover, depressive symptoms were equally associated with functional impairments in all three ethnic groups [7].

Social inequalities, such as differences in socio-economic status, form a possible explanation for mental health inequalities between ethnic groups. Mental health problems have been found to be associated with a less privileged social position [8]. But even though ethnicity and social position are to some extent related, mental health inequalities between ethnic groups cannot be fully attributed to socioeconomic inequalities. The differences in prevalence of mental disorder in the Amsterdam migrant study could for instance not be explained by socioeconomic inequalities [6].

It is conceivable that ethnic mental health inequalities are partly explained by differences in exposure to unfavourable working conditions. In Europe, workers from ethnic minorities are exposed to unfavourable working conditions, such as stressful work, more often than workers from ethnic majorities [9, 10]. However, studies looking at how unfavourable working conditions play a role in explaining ethnic mental health inequalities are scarce. A few studies assess the relationship between perceived work stress and mental ill health in specific occupational groups (see for instance papers on manual workers [11], teachers [12], and mail service employees [13]. One Swedish study looked at differences in mental health as measured by psychotropic medication [14]. This study found that the relationship between working conditions and medication use was not seen in immigrants as opposed to ethnic Swedes. But the prevalence of medication use was very low in immigrants. Whether working conditions contribute to ethnic mental health inequalities still remains to be explored.

Work can be a source of health since work provides income and structure, and is a source of meaning, social interaction and self-respect [15–17]. Accordingly, being employed is considered to promote mental health [18–20]. Work can, however, also pose a threat to the mental health of workers through exposure to unfavourable working conditions [21–24]. In fact, working in jobs with low psychosocial quality poses a mental health risk comparable to that of being unemployed [25, 26]. Unfavourable working conditions may lead to mental disorders through the experience of stress by workers [27, 28].

The link between exposure to unfavourable working conditions and stress has been researched from various theoretical viewpoints such as the Job-Demand Control model [29], and the Effort-reward imbalance model [30]. More recently, the role of recovery opportunities at work has been looked at [31, 32]. Recovery opportunities describe the extent to which workers can recuperate from work, by exerting control over how they plan their workday and by the opportunities to recover after work hours [31]. As such, recovery opportunities have been selected to represent objective working conditions in this study of the role of working conditions in ethnic mental health inequalities. However, stress reactions of workers depend not only on the objective working conditions they are exposed to, but also on how an individual worker appraises that situation [33]. In the present study, perceived work stress is selected to represent the resultant of the objective working condition and the worker’s appraisal. This study aims to investigate whether ethnic mental health inequalities are mediated by a lack of recovery opportunities or perceived work stress. We hypothesize that ethnic groups other than Dutch will have poorer mental health and that this is partially explained by a higher exposure to a lack of recovery opportunities or perceived work stress.

## Methods

### Participants and data collection

Data was provided by the HELIUS study (acronym for Healthy Life in an Urban Setting), a large-scale, multi-ethnic cohort study on health and health care utilization among different ethnic groups living in Amsterdam, the Netherlands. The aims and design of the HELIUS study are described by Stronks et al. [34]. Briefly, participants aged between 18 and 70 years old living in Amsterdam were randomly sampled, and stratified for ethnicity through the municipality register of Amsterdam. This registry contains data on the country of birth of residents and their parents, which are needed to determine ethnicity. Data were collected via a questionnaire and a physical examination. Translated questionnaires were available in English for the Ghanaian and Turkish for Turkish participants. Surinam participants were provided with the Dutch questionnaire, which is their mother tongue. Because most Moroccans are not able to read the official Moroccan language (Moroccan Arabic) and their spoken language (Berber) consists of many different dialects, the questionnaire was not translated in Moroccan. Participants unable to fill in a questionnaire were offered assistance by a trained (ethnically matched) interviewer. For Moroccan interviewers, a phonetic translation of the questionnaire was available. The study protocols were approved by the AMC Ethical Review Board, and all participants provided written informed consent. Baseline data collection of the HELIUS study started in 2011 and is still ongoing. Therefore, definite response rates cannot be calculated yet. At the end of 2014, response rates were estimated at 20 to 40 % with some variations across ethnic groups.

For the current study, baseline questionnaire data of 6472 employed participants, collected up until December 2013, were used. Following the definition of Netherlands Statistics, employment was defined as working at least twelve hours per week. In the Netherlands, the rate of workers with part-time jobs is overall high. Women work in part time jobs more often than men. Men work a mean of 36 h per week while women work a mean of 25 h per week [35]. Working part time is therefore not necessarily the consequence of a mental health problem. Nevertheless, the rate of working part time is higher in people with mental health problems [36].

### Measures

#### Demographic and occupational characteristics

Demographic data retrieved from the baseline questionnaire included sex, age, educational level (categories including ‘no or elementary schooling’; ‘lower vocational or secondary schooling’; ‘intermediate vocational or secondary schooling’; ‘higher vocational schooling or university’), and marital status (categories were categorized into ‘married or living together’; ‘never been married’; ‘divorced or widowed’). Occupational level was classified according to the Dutch Standard Occupational Classification [37] into ‘elementary’, ‘lower’, ‘intermediate’, ‘higher’, or ‘academic’, based on job title, job description and a question on fulfilling an executive function. The number of weekly working hours was assessed in the questionnaire with the following answering options concerning the weekly hours worked: between 12 and 20; between 20 and 32; 32 h or more. Irregularity of working hours was assessed with a single yes or no question: “Does your job require working irregular hours, such as shift work or night shifts?”. For each of the occupational characteristics, workers are asked to rate “their job” without any reference to whether they had a single or multiple jobs.

#### Ethnicity

In HELIUS, a person is defined as being of non-Dutch ethnic origin if she or he fulfils one of two criteria: he or she was born outside the Netherlands and has at least one parent who was born outside the Netherlands (first generation); or he or she was born in the Netherlands but both parents were born outside the Netherlands (second generation) [38]. In the Netherlands, ethnic groups defined by country of birth or ones parents’ country of birth is a widely accepted classification method. It does not, however, align with ethnic identity of individuals in case of different ethnic groups living in the same country of origin [34, 38]. For that reason, in participants of Surinamese origin, ethnic subgroups were classified according to self-reported ethnic origin. Therefore, ethnic background was classified as either Dutch, African Surinamese, South-Asian Surinamese, Indonesian Surinamese, Surinamese with unknown background, Turkish, Ghanaian, Moroccan, or other/undefined.

#### Indicators of working conditions

Two indicators of unfavourable working conditions were included in HELIUS. The first indicator is a work-related recovery opportunities scale, designed to capture the work characteristics allowing workers to recuperate from work effort [31]. Both opportunities for time off the job and aspects of the job design allowing workers to control rest breaks and interruptions during the work day are represented in this nine-item scale. Sample questions are “Can you interrupt your work if you find it necessary to do so?”, “Do you have the possibility of working hours which suit the particular requirements of your private life?”. Answering options are: 3) always; 2) often; 1) sometimes; and never (0). All but two items are reverse coded. The sum score is transformed to a 0–100 score range, with higher scores reflecting few recovery opportunities. Good reliability and good content-, construct-, and criterion-related validity was shown in three samples of workers [31]. Work-related recovery opportunities was dichotomized based on the decile score. Deciles eight to ten were classified as lack of recovery opportunities (coded as 1), based on the finding of Veldhoven et al. [31]. They found that in a heterogeneous population with a comparable mean score (mean score 38 in both the Veldhoven study sample and ours), insufficient recovery after work was especially evident in these upper three deciles. Scores up until the seventh decile were coded 0, representing low exposure to unfavourable working conditions.

Perceived stress at work was assessed with a single question, derived from the INTERHEART study [39]: “How often in the last 12 months have you felt stressed (feeling irritable, filled with anxiety, or having sleeping difficulties) as a consequence of your work or working conditions?”. Answering options were: 1) never; 2) some periods; 3) several periods; or 4) permanent stress. Perceived stress at work was dichotomized into never-some periods (coded ‘0’) versus several periods-permanent stress (coded ‘1’).

#### Mental health indices

Two mental health outcomes were used: generic mental health and depressive symptoms. Generic mental health was assessed using the Mental Component Summary Score (MCS) from the Medical Outcomes Study Short Form 12 (SF-12) [40]. The SF-12 has demonstrated good reliability and validity in previous research [40–43]. Higher scores reflect better mental health. Scores are standardised to United States population norms with the mean score set at 50 (SD 10) following the scoring guideline by Ware [44]. As this scoring method assigns a favourable score to missing items, this methods was only applied to calculate sum scores with one missing item. No sum scores were calculated for scales with more than one item. Using this scoring algorithm, a mean of 51.6 (SD 9.2) was found in the general Dutch population [42]. The sampling frame for this norm population was drawn from the municipal population registry of Amsterdam. No data on the representation of ethnic minorities in this sample was presented.

Depressive symptoms were assessed by means of the Patient Health Questionnaire-9 (PHQ-9).

The PHQ-9 is the nine-item mood module of the PHQ. Each item of the PHQ-9 evaluates the presence of one of the nine *Diagnostic Statistical Manual-IV* criteria of a depressive episode in the past two weeks: (a) depressed mood; (b) anhedonia; (c) trouble sleeping; (d) feeling tired; (e) change in appetite or weight; (f) guilt or worthlessness; (g) trouble concentrating; (h) feeling slowed down or restless; (i) suicidal thoughts. There are four answer categories: 0 (*not at all*), 1 (a *few days*), 2 (*more than half the time*) and 3 (*almost every day*). Depressive symptoms were assessed by the sum score (0–27), with higher scores reflecting more depressive symptoms. If one item was missing, a sum score was calculated by imputing the missing value with the mean of the other eight items. No sum scores were calculated for scales with more than one missing item. Only sum scores were used in this study, but cut-off scores have been reported in previous studies. Scores of 5 to 9, 10 to 14, and 15–27 represent mild, moderate, and severe levels of depression severity, respectively [45]. While the PHQ-9 was designed for use in healthcare settings, the internal consistency and construct validity were found to be good in the general population bases on a German household sample [46]. After comparing ethnic Dutch and ethnic Surinam groups, a Dutch study in a primary care population concluded that overall the PHQ-9 is not culturally biased [47].

Prolonged fatigue was measured with a single question derived from a chronic conditions questionnaire derived from the health monitor of the Public Health Service Amsterdam [48]. Participants were asked whether they had experienced any of a list of diseases and (chronic) health conditions in the previous 12 months. Prolonged fatigue was described as “severe or chronic fatigue”. Possible answers were “no”, “yes, but not diagnosed by a doctor”, and “yes, diagnosed by a doctor”. The “no” category was coded ‘0’, and both “yes” categories were coded ‘1’.

### Analyses

#### Ethnic mental health inequalities and working conditions

First, tests were performed to establish whether ethnic mental health inequalities were found in this study. Due to non-normality of these continuous outcomes, the Kruskal Wallis test was used. Next, we tested differences in the way unfavourable working conditions, lack of recovery opportunities and perceived stress at work, were distributed over the different ethnic groups using the Chi-square test. Finally, the relationship between unfavourable working conditions and mental health outcomes was investigated while controlling for ethnicity using linear regression. To assess whether gender differences are prominent, these analyses were repeated for men and women separately.

#### Mediation analysis

Mediation of the relationship between ethnicity and mental health by exposure to unfavourable working conditions was studied by regression analysis. Linear regression was conducted for generic mental health and depressive symptoms, while adjusting for age and gender. Statistical tests of mediation of unfavourable working conditions in the relationship between ethnicity and mental health were conducted with the bias-corrected bootstrap confidence intervals technique. The procedure advocated by Hayes was used to accommodate the categorical nature of the independent variable (ethnicity) [49, 50]. The ethnic Dutch group was used as reference group. The mediator (working conditions) and outcome variable (mental health) were entered in the model as continuous variables.

To obtain a measure of the extent to which potential mediators attenuate the effect of ethnicity on mental health, uncorrected regression models of ethnicity and mental health were compared to these models after correcting for unfavourable working conditions. The percentage of change in the standardised regression coefficients after adding the potential mediator to the models was calculated. The following formula was used: (b_extended_ –b_basic model_)/(b_basic model_-1) [51].

All analyses were conducted using IBM SPPS 16 Statistical package, for the mediation analyses, a macro was used (available at http://www.afhayes.com/spss-sas-and-mplus-macros-and-code.html).

## Results

### Participants

**Fig. 1:**
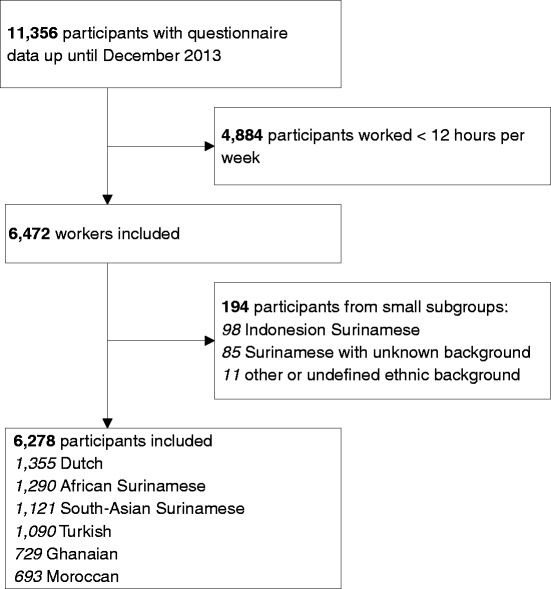
Flowchart of the inclusion of participants in this subset of the Helius study

In Fig. 1 a flowchart of the inclusion process is shown. Of the 11,356 participants with available questionnaire data included in HELIUS up until December 2013, 6472 (57 %) worked at least 12 h per week. The Indonesian Surinamese group (98), the Surinamese with unknown background group (85) and those with another or undefined ethnic background (11) were excluded as these subgroups were too small to be analysed. In the end, 6278 participants were included, these being of Dutch (1355), African Surinamese (1290), South-Asian Surinamese (1121), Turkish (1090), Ghanaian (729), and Moroccan (693) ethnic origin. Table 1 presents the characteristics of these participants.

**Table 1:** Characteristics of working participants HELIUS study, *N* = 6278

Demographic	
Gender, N (%),	
Male	3154 (50)
Female	3124 (50)
Age in years, mean (SD)	43 (12)
Educational level, N (%)	
No or elementary	680 (11)
Lower vocational or secondary	1623 (26)
Intermediate vocational or secondary	1933 (31)
Higher vocational or university	2019 (32)
Marital status, N (%)	
Married or living together	3335 (53)
Never married	2091 (33)
Divorced or widowed	818 (13)
Occupational level, N (%)	
Elementary-low	2544 (41)
Intermediate-academic	3407 (54)
Weekly working hours, N (%)	
12–19	555 (9)
20–32	1154 (18)
>32	4569 (73)
Irregular working hours, N (%)	
No	4866 (79)
Yes	1314 (21)
Ethnicity	
Ethnic background, N (%)	
Dutch	1355 (22)
African Surinamese	1290 (21)
South-Asian Surinamese	1121 (18)
Turkish	1090 (17)
Ghanaian	729 (12)
Moroccan	693 (11)
Migration generation of non-Dutch origin, N (%)	
First	3801 (77)
Second	1122 (23)
Working conditions	
Lack of recovery opportunities, N (%)	
No	4288 (70)
Yes	1849 (30)
Perceived stress at work, N (%)	
Never-sometimes	4966 (83)
Often-always	1009 (17)

Percentages may add up to over a 100 % due to round-off differences

N ranged from 5975 to 6278 due to missing values

Because the recruitment process for the baseline inclusions of the HELIUS study is still ongoing, definite response-analyses cannot yet be done. We performed, however, a preliminary non-response analysis to investigate whether participants to HELIUS differed regarding relevant demographic characteristics from the non-responders. These preliminary analyses showed an overall response rate of about 27.2 % with some variations between the ethnic groups (30.9 % in Dutch, 28.7 % in Surinamese, 37.7 % in Ghanaian, 21.9 % in Turkish, and 22.4 % in Moroccan). The non-responders were on average younger with some overrepresentation of males across all ethnic groups.

### Ethnicity in relation to mental health

Table 2 displays the mental health indices as distributed over the various ethnic groups.

**Table 2:** Mental health indices in relation to ethnicity of working participants HELIUS study

	Generic mental health (MCS scores range from 0–100, higher scores reflect better mental health) *N* = 5551				Depressive symptoms (PHQ scores range from 0–27, higher scores reflect more depressive symptoms) *N* = 5557		
Ethnic group	Mean	SD	Median	Inter Quartile Range	Mean	SD	% scoring ≥ 10
Dutch, total	53.0	6.7	55.0	6.4	3.2	3.2	5
Men	52.5	6.3	55.2	6.4	2.7	2.8	3
Women	53.6	7.0	54.0	6.5	3.7	3.4	6
African Surinamese, total	52.5	8.3	54.7	8.6	3.2	3.8	7
Men	53.6	7.7	55.7	9.1	2.6	3.1	4
Women	51.8	8.6	53.9	9.3	3.6	4.1	9
South-Asian Surinamese, total	50.7	9.5	53.3	10.4	4.2	4.8	13
Men	51.6	9.5	54.0	9.9	3.5	4.3	9
Women	49.8	9.4	52.2	10.4	4.9	5.2	17
Turkish, total	48.7	9.7	50.9	11.9	5.1	5.1	16
Men	50.1	9.1	52.2	10.2	4.5	4.9	13
Women	46.5	10.3	48.3	14.5	6.1	5.4	21
Ghanaian, total	51.8	8.0	53.1	9.9	2.7	3.6	6
Men	52.4	7.5	54.4	8.8	2.2	3.2	4
Women	51.8	8.3	52.3	10.4	3.4	4.0	7
Moroccan, total	50.0	8.9	52.5	9.4	4.5	4.8	13
Men	50.6	8.7	53.0	9.1	4.0	4.6	10
Women	49.3	9.2	51.7	10.7	5.3	5.1	17

Generic mental health (*p* < .001) and depressive symptoms (*p* < .001) differed between (total) ethnic groups, according to the Kruskal Wallis test

Ethnic mental health inequalities were found (Table 2). For both mental health indices, most ethnic minorities show poorer mental health than the ethnic Dutch group in these unadjusted analyses. The only exceptions were that Ghanaian workers, compared to ethnic Dutch workers, report fewer depressive symptoms (2.7 vs. 3.2), and African Surinamese workers show comparable generic mental health (52.5 vs. 53) and depressive symptoms (3.2 vs. 3.2) as ethnic Dutch workers. The Turkish group stands out with the lowest mental health on both mental health indices, followed by Moroccan and South-Asian Surinamese workers.

### Ethnicity in relation to unfavourable working conditions (mediators)

**Fig. 2:**
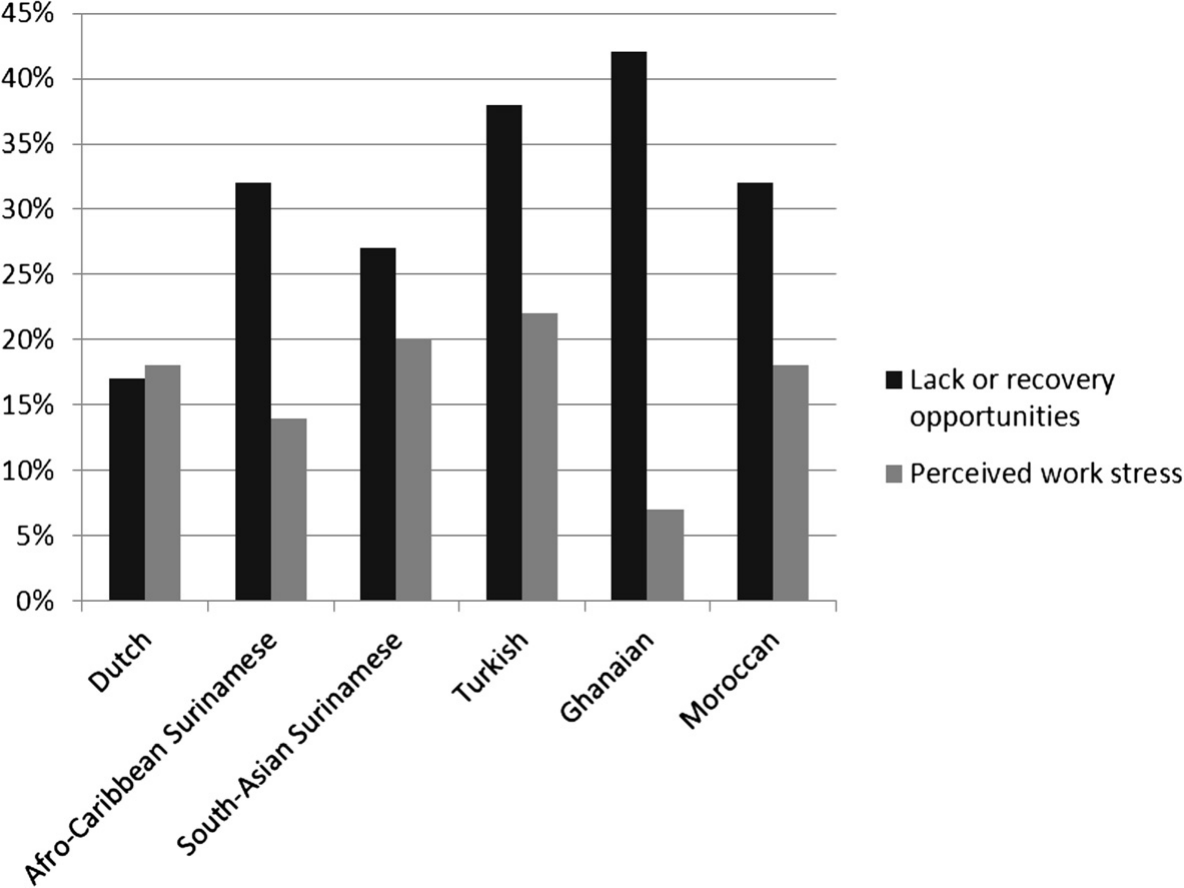
Proportion of workers with unfavourable working conditions for each ethnic group. Ethnic inequalities in lack of recovery opportunities (*p* < .001) and perceived work stress (*p* < .001) were found using the Chi-square test

The proportions of workers with unfavourable working conditions, the so-called mediators, among the different ethnic groups are presented in Fig. 2. Ethnic groups showed statistically significant differences in prevalence of unfavourable working conditions, with all ethnic minority groups having higher lack of recovery opportunities compared to ethnic Dutch workers (17 %). The highest percentage of workers with lack of recovery opportunities was found among Ghanaian workers (42 %), followed by Turkish (38 %), African Surinamese (32 %), Moroccan (32 %), and South-Asian Surinamese workers (27 %). The distribution of perceived work stress among ethnic groups shows a different pattern. The lowest proportion of perceived work stress can be found in the Ghanaian group (7 %), rather than in the ethnic Dutch group (18 %). African Surinamese workers also reported work stress less often than Dutch workers (14 %). Compared to ethnic Dutch workers, perceived work stress was higher only in Turkish (22 %) and South-Asian Surinamese workers (20 %).

### Unfavourable working conditions (mediators) in relation to mental health

While controlling for ethnicity, lack of recovery opportunities was associated with poorer mental health indicated by both mental health outcomes (generic mental health: B −1.88, CI −2.34 to −1.42; and depressive symptoms: B 1.04, CI 0.81 to 1.27). Perceived work stress was also associated with poorer mental health outcomes, with stronger associations compared to recovery opportunities (generic mental health: B −8.26, CI −8.79 to −7.72; depressive symptoms: B 4.56, CI 4.29 to 4.82). These relationships were all fairly similar for men and women separately. All tested associations showed the same pattern; the associations were less strong in women but remained statistically significant. One exception was the model with recovery opportunity and depressive symptoms, where the B coefficient for men was .92 and 1.01 for women, indicating a stronger association in women.

### Mediation of the relation between ethnicity and mental health by unfavourable working conditions

For both mental health outcomes, a basic regression model assessed the relationship between ethnicity and mental health, adjusted for age and gender. In the next two separate models, a lack of recovery opportunities and perceived stress at work indicators were added. Tables 3, 4, 5 and 6 show the regression models with b coefficients for each ethnic group compared to the Dutch reference group.

**Table 3:** Linear regression of generic mental health^a^ explained by ethnicity, with recovery opportunities as potential mediator

Recovery opportunities Models:	1: Ethnicity + age and gender		2: Ethnicity + recovery opportunities + age and gender		Attenuation %^b^	Mediation test^c^	95 % CI (bootstrap)
	b	95 % CI	b	95 % CI			
Dutch (reference)	-	-	-	-	-	-	-
African Surinamese	−0.40	(−1.02 to 0.23)	−0.02	(−0.65 to 0.61)	-	**−0.41**	(−0.54 to −0.30)
South-Asian Surinamese	**−2.33**	(−2.98 to −1.66)	**−2.10**	(−2.7 to −1.41)	−10 %	**−0.32**	(−0.44 to −0.22)
Turkish	**−4.25**	(−4.93 to −3.58)	**−3.61**	(−4.3 to −2.93)	−15 %	**−0.63**	(−0.80 to −0.48)
Ghanaian	**−1.35**	(−2.10 to −0.60)	−0.67	(−1.43 to 0.09)	−50 %	**−0.74**	(−0.93 to −0.57)
Moroccan	−**2.81**	(−3.58 to −2.04)	**−2.35**	(−3.12 to −1.57)	−16 %	**−0.44**	(−0.58 to −0.31)

^a^Scores in generic mental health range from 11 to 70, higher scores reflect better generic health

^b^% Change in B calculated as (B_ethnicity+workcondition_-B_ethnicity_)/(B_ethnicity_); only for models where the ethnic group showed a statistically significant higher risk of mental health problems and the mediation test for that ethnic group was statistically significant. Negative signs (−) are used for changes towards non-significance (zero B)

^c^Bold printed figures represent statistically significant mediation for that ethnic group

**Table 4:** Linear regression of generic mental health^a^ explained by ethnicity, with work stress as potential mediator

Works stress Models:	1: Ethnicity + age and gender		2: Ethnicity + work stress + age and gender		Attenuation %^b^	Mediation test^c^	95 % CI (bootstrap)
	b	95 % CI	b	95 % CI			
Dutch (reference)	-	-	-	-	-	-	-
African Surinamese	−0.40	(−1.02 to 0.23)	**−1.15**	(−1.75 to −0.55)	188 %	**0.81**	(0.58 to 1.05)
South-Asian Surinamese	**−2.33**	(−2.98 to −1.66)	**−2.47**	(−3.09 to −1.84)	-	0.12	(−0.14 to 0.37)
Turkish	**−4.25**	(−4.93 to −3.58)	**−4.37**	(−5.00 to −3.73)	-	0.14	(−0.12 to 0.40)
Ghanaian	**−1.35**	(−2.10 to −0.60)	**−2.90**	(−3.61 to −2.18)	115 %	**1.61**	(1.33 to 1.91)
Moroccan	−**2.81**	(−3.58 to −2.04)	**−3.41**	(−4.14 to −2.67)	21 %	**0.56**	(0.26 to 0.86)

^a^Scores in generic mental health range from 11 to 70, higher scores reflect better generic health

^b^% Change in B calculated as (B_ethnicity+workcondition_-B_ethnicity_)/(B_ethnicity_); only for models where the ethnic group showed a statistically significant higher risk of mental health problems and the mediation test for that ethnic group was statistically significant. Negative signs (−) are used for changes towards non-significance (zero B)

^c^Bold printed figures represent statistically significant mediation for that ethnic group

**Table 5:** Linear regression of depressive symptoms^a^ explained by ethnicity, with recovery opportunities as potential mediator

Recovery opportunities Models:	1: Ethnicity + age and gender		2: Ethnicity + recovery opportunities + age and gender		Attenuation %^b^	Mediation test^c^	95 % CI (bootstrap)
	b	95 % CI	b	95 % CI			
Dutch (reference)	-	-	-	-	-	-	-
African Surinamese	0.01	(−0.30 to 0.32)	−0.22	(−0.53 to 0.09)	-	**0.22**	(0.17 to 0.28)
South-Asian Surinamese	**1.07**	(0.75 to 1.39)	**0.92**	(0.60 to 1.25)	−14 %	**0.17**	(0.11 to 0.23)
Turkish	**1.82**	(1.49 to 2.16)	**1.49**	(1.16 to 1.83)	−18 %	**0.34**	(0.26 to 0.42)
Ghanaian	−0.34	(−0.71 to 0.03)	**−0.75**	(−1.12 to −0.37)	-	**0.40**	(0.31 to 0.49)
Moroccan	**1.23**	(0.84 to 1.61)	**0.96**	(0.58 to 1.34)	−22 %	**0.24**	(0.17 to 0.32)

^a^Depressive symptoms scores range from 0 to 27, higher scores reflect more depressive symptoms

^b^% Change in B calculated as (B_ethnicity+workcondition_-B_ethnicity_)/(B_ethnicity_); only for models where the mediation test for that ethnic group showed a statistically significant higher risk of mental health problems and was statistically significant. Negative signs (−) are used for changes towards non-significance (zero B)

^c^Bold printed figures represent statistically significant mediation for that ethnic group

**Table 6:** Linear regression of depressive symptoms^a^ explained by ethnicity, with work stress as potential mediator

Works stress Models:	1: Ethnicity + age and gender		2: Ethnicity + work stress + age and gender		Attenuation %^b^	Mediation test^c^	95 % CI (bootstrap)
	b	95 % CI	b	95 % CI			
Dutch (reference)	-	-	-	-		-	-
African Surinamese	0.01	(−0.30 to 0.32)	**0.47**	(0.18 to 0.76)	-	**−0.47**	(−0.61 to −0.34)
South-Asian Surinamese	**1.07**	(0.75 to 1.39)	**1.17**	(0.87 to 1.47)	-	−0.07	(−0.21 to 0.08)
Turkish	**1.82**	(1.49 to 2.16)	**1.91**	(1.60 to 2.21)	-	−0.08	(−0.22 to 0.08)
Ghanaian	−0.34	(−0.71 to 0.03)	**0.50**	(0.15 to 0.84)	-	**−0.90**	(−1.07 to −0.75)
Moroccan	**1.23**	(0.84 to 1.61)	**1.53**	(1.18 to 1.88)	24 %	**−0.30**	(−0.47 to −0.13)

^a^Depressive symptoms scores range from 0 to 27, higher scores reflect more depressive symptoms

^b^% Change in B calculated as (B_ethnicity+workcondition_-B_ethnicity_)/(B_ethnicity_); only for models where the mediation test for that ethnic group showed a statistically significant higher risk of mental health problems and was statistically significant. Negative signs (−) are used for changes towards non-significance (zero B)

^c^Bold printed figures represent statistically significant mediation for that ethnic group

The attenuation of the associations (in%) is represented only for models where a higher risk of mental health problems was present and the mediation test for that ethnic group was statistically significant. The analyses of generic mental health (Tables 3 and 4) show that, when adjusted for age and gender, all but the African Surinamese workers, have less favourable mental health when compared to the Dutch reference group.

Lack of recovery opportunities mediated the relationship between ethnicity and generic mental health in all groups. In each of these groups, taking recovery opportunities into account attenuated the associations, changing the association towards being more favourable. The extent of the attenuation of the ethnicity-generic mental health relationship differed between groups. In Ghanaian workers, a change in b coefficient of 50 % was observed, in Moroccan workers this figure was 16 %, in Turkish workers 15 %, and in South-Asian Surinamese workers 10 %. A different pattern emerged when work stress was taken into account. When taking perceived work stress into account, the adjusted association of ethnic group with worse generic mental health increased in Ghanaian and Moroccan workers. In Ghanaian workers, this was related to the fact that the percentage of people reporting work stress in this groups was lower than in the ethnic Dutch.

The analyses of depressive symptoms (Tables 5 and 6) show that, when adjusted for age and gender, all but the African Surinamese and Ghanaian workers, have more depressive symptoms when compared to the Dutch reference group.

Recovery opportunities mediated the association of ethnic groups with depressive symptoms. In all ethnic groups, the association with depressive symptoms changed towards being more favourable. In the other groups, a change in b coefficient was observed of 14, 18, and 22 % for South-Asian Surinamese, Turkish, and Moroccan workers, respectively. The mediation patterns for work stress in relation to depressive symptoms showed that, as with generic mental health, no mediation effect of work stress was observed for South-Asian Surinamese and Turkish workers. For African Surinamese, Ghanaian and Moroccan workers, adding work stress to the model, increases the risk of depressive symptoms. It should be noted that Ghanaian workers had fewer depressive symptoms in the uncorrected model and African Surinamese did not differ from the ethnic Dutch group. As work stress in these groups was lower than in the ethnic Dutch group, controlling for this factor led to a higher risk of depressive symptoms.

## Discussion

In this large cross-sectional sample of workers from various ethnic groups, we found ethnic mental health inequalities, with most ethnic minorities having increased risks of mental health problems when compared to the ethnic Dutch group. After adjusting for age and gender, all but the African Surinamese workers, have less favourable mental health when compared to the Dutch reference group. And all but the African Surinamese and Ghanaian workers, have more depressive symptoms when compared to the Dutch reference group. This study examined whether increased mental health risks for ethnic minorities could be partly explained by exposure to unfavourable working conditions. All ethnic minority groups had lack of recovery opportunities at work more often, as compared to ethnic Dutch workers. This lack of recovery opportunities mediated the relationship between ethnic group and a higher risk of mental health problems. Perceived work stress was only higher in the Turkish and South-Asian Surinamese groups, but mediation effects of the relationship between ethnicity and mental health were not found in these groups. In Ghanaian and African Surinamese groups, work stress was less prevalent than in Dutch workers. Taking work stress into account thus increased the risk of mental health problems in these ethnic groups. Our findings therefore suggests that perceived work stress does not contribute to the explanation of higher mental health risks in ethnic minorities.

Our findings that mental health problems are most prominent in Turkish and Moroccan workers are in line with previous Dutch studies among immigrants [6]. That study did not differentiate between various Surinamese ethnic groups, thereby impeding a further comparison of ethnic groups. Drawing on data from the HELIUS study can be considered a strength of this study on ethnic mental health inequalities as this ensured a considerable sample size including a variety of ethnic groups. Furthermore, ethnicity was carefully defined, sampled and recorded in this study, thereby reducing imprecision in our exploration of ethnic mental health disparities. It should be noted that ethnicity was defined based on the country of birth of the workers and that of their parents. This implies that ethnic mental health inequalities for first generation immigrants may partly be explained by experienced in their host country.

Several methodological aspects of our study deserve further consideration. First and foremost, the cross-sectional nature of the data should be acknowledged. Causal inferences of our findings cannot be made. In theory, mental health status may have influenced the reporting of working conditions as these are measured by self-report [52]. While this possibility cannot be ignored, reporting on recovery opportunities during a workday is rather factual and perhaps less susceptible to mood states than measures of, for instance, social relations at the workplace. Perceived work stress may be more susceptible to mood states as this concept is more closely related to mental health outcomes. However, our data did not reveal such an effect as perceived work stress did not contribute to the explanation of higher mental health risks in ethnic minorities.

A second methodological aspect of our study concerns the use of self-report measures, rather than clinical interviews, for measuring depression. Whereas the PHQ-9 is well validated to detect and monitor depression [45], it should be noted that we chose to use depressive symptoms, and not a clinically diagnosed depressive disorder, as an indicator of mental health. Therefore, we cannot extend our conclusions on mental health to clinically diagnosed depressive disorders.

Thirdly, our single-item used to measure perceived work stress can be considered rather crude. This instruments was used in prior studies where it was found to be predictive of an increased risk of acute myocardial infarction [39]. The effects were found to be consistent across regions and in different ethnic groups. However, work stress was a categorical variable, which is prone to misclassifications. Misclassifications in mediator variables may lead to an underestimation of the mediation effect. All in all, we cannot exclude that more elaborate measures of perceived work stress would have contributed more to the explanation of ethnic mental health disparities.

Finally, the clinical relevance of the mental health disparities between ethnic groups could not be determined in our study. The MCS was used to measure generic mental health, but this measure does not have a validated cut-off point for low mental health in the Dutch population. In an Australian population, a <50 cut-off was advised to screen for any common mental disorder [43]. The mean of the Turkish workers was below that cut-off (48.7). The depressive symptoms scores (PHQ-9) ranged from 2.7 (Ghanaian) to 5.1 (Turkish). The cut-off score for mild depression severity is five, meaning that the mean score for Turkish workers indicates mild depression, giving some indication of the clinical relevance of the disparities.

Our findings call for further research into the origin of mental health disparities among ethnic groups. We were only able to include a limited number of measures on working conditions. One possible direction for future research would be to look at other psychosocial working conditions. Conditions such as job demands, a lack of job control, and an effort-reward imbalance (e.g. [53] and [54]) and workplace bullying (e.g. [55]) have been found to influence mental health in the general working population.

Research into more ethnic-specific experiences at the workplace, such as unfair treatment and workplace discrimination, may prove to be another fruitful direction for future research. Ethnicity has been linked to unfavourable working conditions in earlier studies [9], But since working conditions are usually measured by self-report, perception and circumstances cannot be disentangled. One interesting study shedding some light on this issue was conducted in the US. Workers within one occupation (nursing) were classified as working in higher- and lower-skilled jobs and findings showed that within the same occupational class, black workers were 2.9 times more likely to report job strain compared to white workers. The authors put workplace racial/ethnic discrimination forward as one of the explanations [56]. The notion that discrimination at the workplace may be an ethnic-specific risk factor for poor mental health was also found by Bhui and colleagues [57]. They reported an association between common mental disorders and the experience of racial insults and perception of unfair treatment at work. Future studies incorporating generic working conditions such as recovery opportunities, and ethnic-specific experiences such as discrimination may shed more light on the causes of mental health problems in ethnic minorities.

## Conclusions

This study shows that all ethnic minorities, with the exception of the Ghanaian and African Surinamese workers, have an increased risk of both poorer mental health and higher depressive symptoms. These ethnic mental health inequalities are partly accounted for by a lack of recovery opportunities at work. Perceived work stress does not contribute to the explanation of these inequalities. This may imply that workplace prevention targeting recovery opportunities have the potential to reduce ethnic inequalities, but ethnic-specific experiences at the workplace need to be further explored.

## Abbreviations

Helius: Healthy life in an urban setting
SF-12: Medical outcomes study short form 12
PHQ-9: Patient health questionnaire-9
